# Applying the multiple object juggling task to measure the attention of athletes: Evidence from female soccer

**DOI:** 10.1097/MD.0000000000037113

**Published:** 2023-02-02

**Authors:** Qian Su, Feng Wang, Jingcheng Li, Qiang Dai, Baokun Li

**Affiliations:** a School of Kinesiology and Health, Capital University of Physical Education and Sports, Beijing, China; b School of Physical Education, Dankook University, Yongin City, Korea; c Department of Physical Education, Tongren Nursery Normal College, Tongren, China; d Department of Psychological Counseling, Paichai University, Daejeon, Korea.

**Keywords:** added condition, dynamic condition, female soccer player, fixed condition, multiple object juggling (MOJ), multiple object tracking (MOT)

## Abstract

The purpose of this study is to investigate whether the presentation of targets can affect the performance of multiple object tracking and whether the difference between female soccer players and female college students is regulated by the presentation of targets. We enlisted a group of 20 Chinese female soccer players and another group of 20 non-players to complete a multiple object juggling (MOJ) task. The mean age was 20.24 ± 1.61 years in the athletes group and 21.35 ± 1.93 years in the non-athletes group. Accuracy was analyzed to examine the disparity between soccer players and non-players, as well as the disparity between 3 presentation conditions for targets (fixed, added, and dynamic). Regarding the MOJ task, female soccer players did not outperform non-players (F = 1.84, 95% CI [–1.14 to 6.02], *P* = .27). The performance of tracking in fixed conditions was superior to that in added and dynamic conditions (MD = 10.33%, 95% CI [4.93 to 15.71], *P* < .001; MD = 9.82%, 95% CI [4.43 to 15.21], *P* < .001). The tracking accuracy of female soccer players was significantly higher than non-players in dynamic condition (F = 7.26, 95% CI [2.19 to 14.59], *P* = .01). According to the findings, experts who specialize in team sports tend to exhibit a greater attention advantage in areas that are pertinent to their field of expertise. For future studies, it will be necessary to employ MOT conditions that are more representative of sport-specific characteristics to strengthen the task ecological validity.

## 1. Introduction

The realization that the capacity for anticipation as well as prompt and accurate decision-making is essential to high-level performance in many sports has sparked interest over the past few decades.^[1,2]^ The capacity to access and process vast quantities of information is of crucial importance. In soccer, for instance, players must track the ball while observing teammates and opponents in order to collect information for decision-making. Attention is a fundamental aspect of decision-making.^[3]^ It facilitates us to monitor objects or regions of visual space and extract data for reporting or storage.^[4]^ The association between visual attention and performance in team ball sports is broadly acknowledged.^[5–9]^ Numerous studies have shown that enhancing the decision-making ability of elite athletes through attention training.^[10–13]^

A significant proportion of empirical research in the field of visual attention has centered on concrete paradigms, such as visual short-term memory, attention tracking, and change blindness, among others.^[14]^ In recent years, researchers have utilized the multiple object tracking (MOT) paradigm^[15]^ to examine the relationship between visual attention and athletic expertise.^[16–19]^

An extensive number of studies reporting on visual tracking performance in sports have focused on the accuracy and reaction time in typical MOT task in laboratory settings. These studies has attempted to investigate differences in visual tracking performance between players at different level and experience. For instance, Zhang et al (2009) examined the difference between volleyball athletes and non-athletes on the performance of a MOT task, the number of distractors and the color and form of the targets were manipulated in experiment. The result showed that the overall reaction time was significantly shorter for volleyball athletes, and they found there was no difference in response error rate. A reason for this is because great number of distractors may have promoted participants’ ability for selective attention to the targets.

Attentional load is particularly critical in affecting the performance of multiple-object tracking for athletes. Recently, Qiu et al (2018) assessed elite, intermediate basketball players and non-athletes on MOT task under 3 attentional load conditions (two, 3 and 4 targets). Although elite players displayed better tracking performance at higher attentional load conditions, no differences were observed among the 3 groups when tracking 2 targets. The results support the current consensus that the effects of expertise in team ball sports could transfer to non-sport-specific attention task. They found that these transfer effects to general cognitive functions occur only in elite athletes with extensive training under higher attentional load. In summary, the aforementioned studies demonstrated that elite athletes possess superior perceptual-cognitive skills compared to intermediate athletes and non-athletes.

Only a few studies have attempted to understand how visual tracking ability in team sports vary as a consequence of different conditions. For example, the study by Vu et al (2022) was the only one to investigated the influence of different scenario types on the visual tracking performance of soccer compared to non-soccer players. Surprisingly, although the soccer players had higher tracking accuracy than non-players, they did not show better visual tracking performance on soccer-specific than on pseudo-random trajectories. The reason for this result may be that the advantage of soccer players in visual tracking task is related to the mechanisms underlying the acquisition of information about the dynamics the scene, independent of its content.^[19]^ Although the study attempted to design an experimental task that was more representative of a real soccer scene, they used a virtual reality-based visual tracking task, in doing so, arguably failed to reflected the actual ability of team players to track changing teammates and opponents on a real field.

While previous research improved our understanding of the behavior and performance of athletes during a visual tracking task. Most of these studies used the typical MOT paradigm, in which all items were presented at once and did not change during the tracking phase. In real-world tracking, it is uncommon for the set of tracked items to be identified all at once and remain unchanged throughout the task.^[20]^ When tracking objects, it is necessary to modify the tracking targets constantly and frequently add and remove items from the tracked set. Thus, we argue that the visual attention task that tracked set changes during a single tracking episode may be a more accurate predictor of performance. However, the underlying mechanisms of how athletes process visual information in motion when keep track of changing multiple teammates and opponents are not well known.

In attempt to bridge this gap in research, Wolf et al (2007) developed a new experimental paradigm to examine tracking ability, namely the multiple objects juggling (MOJ) task. Twelve observers were recruited from community, they were required to track 2 to 4 moving objects on a computer screen under 3 conditions (fixed, added, and dynamic). In the fixed condition, participants completed a standard MOT task. In the additional condition, targets were not identified at the start of each tracking episode, but were instead marked as targets sequentially, after all of the objects began to move.^[20]^ In the dynamic condition, tracked targets could be subtracted in addition to being added. At the conclusion of the tracking episode, the objects stopped moving and turned yellow, and the participants were required to click on the target objects until they were all located, tracking accuracy is the dependent variable in a MOJ task. Wolf et al (2007) found participants’ tracking accuracy was high in all 3 conditions (more than 85%), but did not differ reliably between conditions. Such results indicated that adding and subtracting items from the tracked set does not pose a dramatic challenge to the processes supporting MOT. In contrast to a typical MOT task, the MOJ task has the advantage of tracking a set of dynamically changing targets, as might be the case in a real-world tracking task.

In soccer, athletes should change the tracking target according to the actual situation on the field, this tracking process were similar with the MOJ task. To our knowledge, no study has examined the visual tracking behaviors of female soccer players in dynamic changing situations. Consequently, the purpose of this article was to compare the visual tracking ability of professional female soccer players with that of age- and gender-matched non-players using the MOJ task. A group of elite female soccer players, and a group of non-athletes were recruited to perform an MOJ task in 3 conditions (fixed, added and dynamic). The primary objective was to compare the visual performance of female soccer players and non-athletes tracking a dynamically evolving set of targets over time. Tracking accuracies were analyzed to examined the performance of athletes at different levels in the MOJ task. On the basis of prior research, we hypothesized that female soccer players would exhibit superior MOJ task performance in 3 conditions. Moreover, we hypothesized that the sports expertise effects in terms of dynamic were modulated by the presentation of the targets.

## 2. Materials and methods

### 2.1. Participates

The group of athletes consisted of twenty female soccer players (mean age: 20.24 ± 1.61 years, range: 18–24 years old), Soccer players were recruited from a Chinese women football second division league club. The athletes had trained for at least 6 years, with a minimum of 5 90-minute sessions per week. The non-athletes group consisted of twenty female students from the Capital University of Physical Education and Sports (mean age: 21.35 ± 1.93 years, range: 18–24 years old) without experience in professional soccer training or any other sports, all of whom were right-handed and reported normal or corrected-to-normal vision. They were instructed to get plenty of sleep the night before they took part in the study by not staying up too late. Additionally, they were cautioned against engaging in strenuous physical activity so that they would be in peak physical condition for the experiment. The experimental protocol was carried out in accordance with the guiding principles expressed in the Declaration of Helsinki. The experimental protocol was approved by the regional ethics committee of the Capital University of Physical Education and Sports (No. 2023A059). Before beginning the experiment, all participants provided written informed consent and were compensated for their time.

## 3. Apparatus

The stimulus display and experimental control were performed by MATLAB vision R2007b (The Mathworks Inc., Natick, MA) and the Psychophysics Toolbox, operating on a Lenovo PC with a 14-inch monitor with a display resolution of 1920 × 1280 pixel (px) and a refresh rate of 60HZ.

## 4. Stimuli

The stimuli consisted of 8 identical gray circles, each with a 2° visual angle diameter and a 0.25° thick yellow board against a black background. The objects were contained within a square with a 27.25° outline. The objects began each tracking episode in a 15° diameter circle formation. The circles moved approximately 5° per second along a linear path, bouncing off the edge of the display frame and each other.

## 5. Experiment development

This experiment developed predicated on the regular MOJ task, with several modifications made to render the tasks more applicable to the current investigation. Participants were instructed to sit at a table with a chair and conducted the experiment with their chin and forehead supported by a chinrest 0.57 m from the screen. The response was collected by using the left mouse button. The participants were instructed to respond with their dominant hand. Before the experiment, participants were given written instructions outlining the general procedure and trial sequence. At the beginning of each trial, they were instructed to recognize the location of the flashing targets and keep track of them during the movement phase.

**Figure 1.**
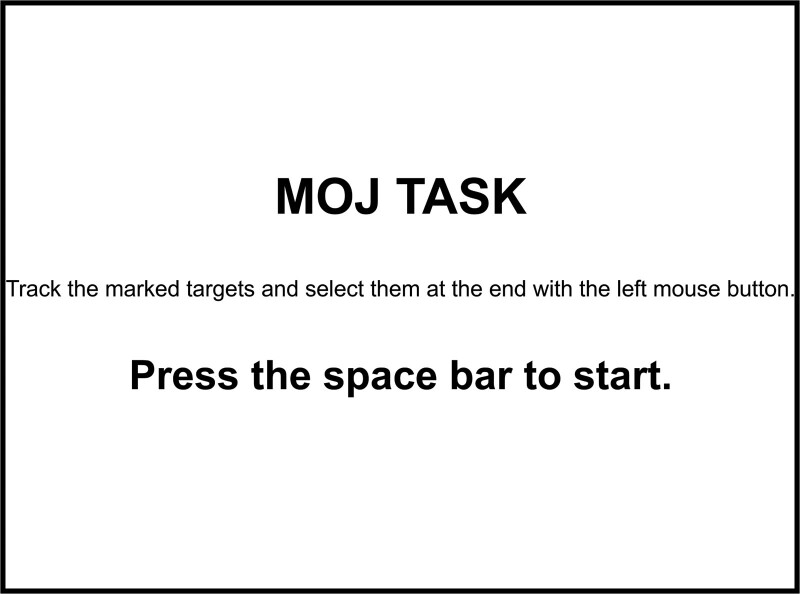
Experimental flow chart. Participants are presented with a start screen and must press the mouse button to continue (A). At the beginning of the trail, a large red “+” appeared in the center of the screen for 1000ms (B). Stimulus materials used in the formal experiment, which including 3 types of targets presentations (C). A feedback screen is presented, and the experiment notes the accuracy of the trail (D).

In each of the 3 experimental conditions, the primary objective was to track the 4 target objects. Participants were required to complete 5 practice trials and 30 experimental trials for each condition. After each condition, participants were invited to take a 2-minute break. Each trial lasted approximately 30 seconds, and each participant spent approximately 50 minutes to complete the experiment. A flow chart of the experiment design is presented in Figure 1.

**Figure 2.**
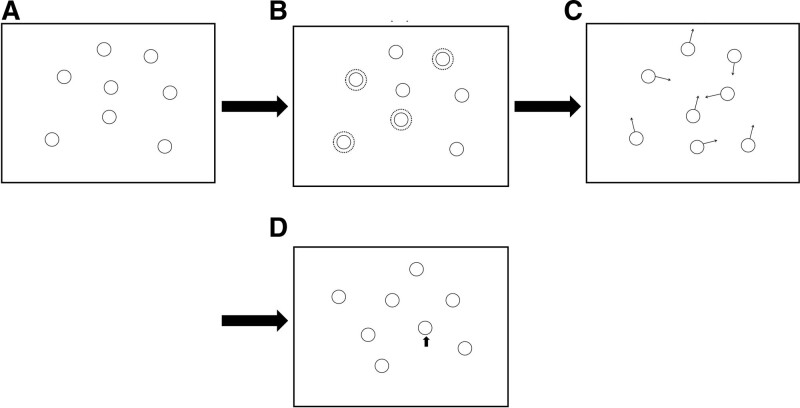
Stimuli and procedure used in the fixed condition. Participants are presented with an array of of identify disks on a background (A). 4 targets were designated at the start of a trail (B). they move around unpredictably among identical non-targets for 10 s (C). during the testing phase, the participants had to answer which of the 4 objects were targets (D).

In accordance with the experimental design, the marking techniques for tracked objects differ across the 3 conditions. In the fixed condition, participants were asked to complete a standard MOT tracking task. At the start of each trial, a fixation cross appeared in the middle of the screen. The fixation cross disappeared after a delay of 1000 ms, and 8 items were presented. Four of the 8 objects were designated as targets by flashing them 5 times with the following pattern: 300msc on, 300msc off. Then, all objects began to move with a speed of 5 degrees per second for 7000 msc. At the conclusion of the tracking episode, the objects stopped moving and turned solid yellow. A mouse cursor appeared in the center of the screen, and the observer was instructed to click on the target object until all of the targets were located as promptly as possible. A flow chart of the fixed condition is presented in Figure 2.

**Figure 3.**
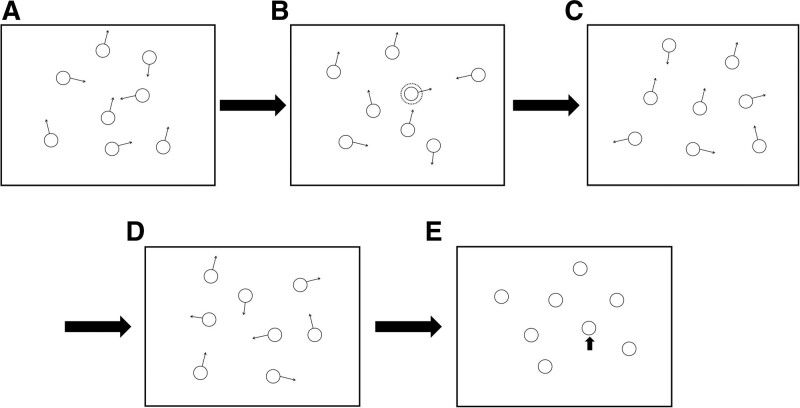
Stimuli and procedure used in the added condition. Participants are presented with a start screen and must press the mouse button to continue. All 8 objects begin to move simultaneously at the beginning of the tracking episode for 1000ms (A). One moving object will marked as target by flashing for 500ms (B). Then the flashing disappeared and all stimuli begin to move independently for 500ms. This sequence is repeated (C). Until 4 targets were marked as targets (D). During the testing phase, the participants had to answer which of the 4 objects were targets (E).

**Figure 4.**
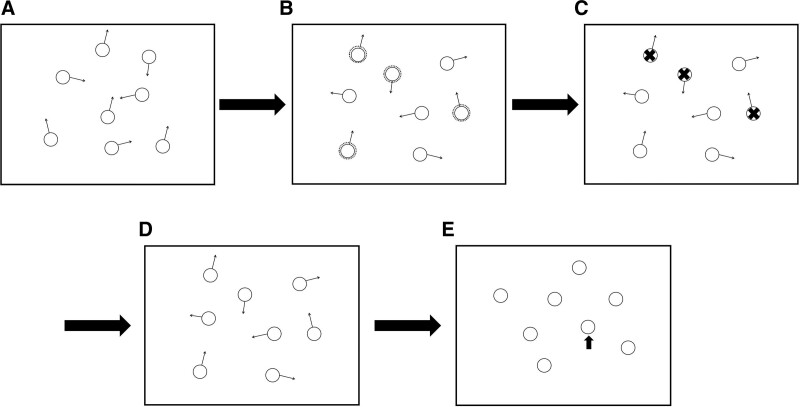
Stimuli and procedure used in the dynamic condition. In this condition, disks could be subtracted from the tracking set, as well as added. The add instruction was the same as in added condition, marked objects as targets by flashing for 1000ms. The subtract instruction was a large red X, placed over a member of the tracked set as it moved. Participants are presented with a start screen and must press the mouse button to continue. All 8 objects begin to move simultaneously at the beginning of the tracking episode for 1000ms (A). Add task: we added targets by flashing objects for 1000ms (B). Subtract task: some large red X were placed over a member of the tracked set as it moved for 1000ms (C). This sequence is repeated over the course of 6 seconds, and then all objects move independently for 10 seconds (D). During the testing phase, the participants had to answer which of the 4 objects were targets (E).

In the add condition, all 8 objects begin to move simultaneously at the beginning of the tracking episode, followed by the sequential marking of 4 disks as targets within 6 seconds. Regardless of how disks were “added” or “subtracted” from the tracked set, observers always had 4 targets to click on at the conclusion of the tracking episode in the dynamic condition. The flow charts of the added and dynamic condition are presented in Figures 3 and 4.

## 6. Experiment design

A mixed design was employed, with a group (expert, novice) serving as the between-subject factor and condition (fixed, added, dynamic) representing the within-subject variable. The outcome measures were performance (% correct).

## 7. Data analysis

The data was recorded and collected by MATLAB 2017b. We used SPSS statistics Version 20.0 for the statistical analyses. In the tracking experiment, a mixed experimental design of 2 (group) × 3 (condition) was used. A linear mixed effect model was used to observe the between-subject effect of the group of participants (female soccer players, non-players), the within-subject effect of experimental condition (fixed, added and dynamic) and their interaction effect on tracking performance. Using a random intercept to account for the repeated measures of each participant. Further analyses were conducted using the sample effects test for any significant interaction. An α-level of 0.05 was set as the level of significance for statistical comparisons.

## 8. Results

The participants’ tracking accuracy averaged 93.85 ± 4.34% in fixed condition, 83.52 ± 7.38% in added condition, 84.91 ± 7.91% in dynamic condition. Group performance by condition are presented in Figure 3. The LMM on visual tracking performance reveled a main significant effect of condition, F = 13.98, 95%CI [–8.30 to –1.36], *P* < .001, indicating that the tracking accuracy of different type of target presentation condition is not the same. Moreover, we found that the tracking accuracy of the subjects in fixed condition was significantly higher than that in added or dynamic condition (MD = 10.33%, 95% CI [4.93 to 15.71], *P* < .001; MD = 9.82%, 95% CI [4.43 to 15.21], *P* < .001), whereas the accuracy difference between the added and dynamic conditions was not statistically significant (MD=–0.50%, 95% CI [–5.89 to 4.89], *P* = .60). However, there was no significant main effect of group (F = 1.84, 95% CI [–1.14 to 6.02], *P* = .27).

**Figure 5.**
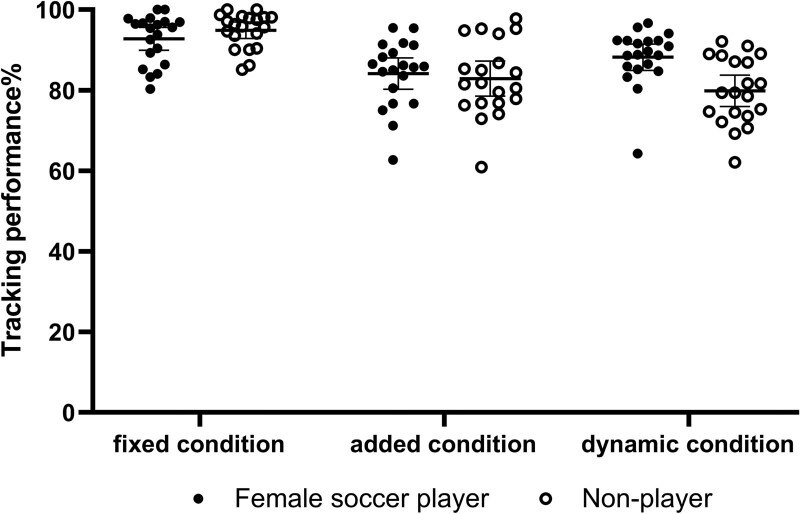
Behavior performance as a function of the target present condition.

Linear mixed effect model revealed a marginal significant interaction between group and target present condition (F = 3.00, 95% CI [0.63 to 9.69], *P* = .06). The results of a simple effect test showed that there was no significant difference between female soccer players and non-players in fixed (F = 0.47, 95% CI [–8.33 to 4.07], *P* = .50) and added condition (F = 0.73, 95% CI [–5.14 to 7.26], *P* = .73), there was a significant difference in tracking accuracy between female soccer players and non-players in dynamic condition (F = 7.26, 95% CI [2.19 to 14.59], *P* = .01), the accuracy of female soccer players was greater than that of non-players (MD = 8.39, see Figure 5).

## 9. Discussion

This study aimed to compare the performance of women soccer experts and novices in juggling multiple objects under 3 conditions: fixed, added, and dynamic. The results indicate that: there was no significant difference between female soccer players and non-players; tracking performance in the fixed condition was superior to that in the added and dynamic conditions; and the tracking accuracy of female soccer player was significantly higher than non-player in dynamic condition.

The primary objective of this study is to investigate the group effect and determine whether the player group differs from the non-player group. Surprisingly, the experimental findings contradict the hypothesis. Specifically, we discovered that there was no statistically significant difference in accuracy between female soccer players and non-players, which is consistent with some earlier findings.^[18,21,22]^ According to flexible-resource theory, tracking is mediated by a limited attentional resource that is distributed among the targets,^[23,24]^ it is possible to strengthen the ability of participants to track multiple targets at a slow speed.^[23]^ Non-players have sufficient attentional resources to allocate to each target when tracking 4 slow-moving targets (5°/s) in the present study. This may reach a high level of tracking accuracy which is the same as the female soccer players. This explanation is supported by the fact that participants completed the MOT task at speed of 5°/s and no significant correlation was found between basketball players and non-athletic college students.^[21]^ on the other hand, it could be possible that the characteristics of MOJ task and the soccer-specific are quite different, the result can not reflect the advantage of soccer players’ special attention. Williams et al found that soccer players were more accurate in recognized structured trails only, no significant difference were found between in unstructured condition.^[25]^ Future research should examine the ability to track objects with identity information in real situation.

We found that the tracking performance in fixed condition was superior to that in the added and dynamic condition, suggesting that adding or subtracting items from the tracked set can affect the MOT performance, which is inconsistent with prior findings.^[20]^ Wolf et al (2007) evaluated performance on the MOJ task, and the results revealed no statistically significant difference between conditions. The reason for the inconsistency may be caused by the difference in design of the experiments. The objects moving at the speed of 12°/s in the Wolfe study, but the objects moving at the speed of 5°/s in our experiment. A slower speed of object movement may increase the attention costs incurred by subjects from adding or subtracting items from tracking set. According to perceptual load hypothesis,^[26]^ one way in which the attentional system negotiates cognitive overload is to process less extraneous information in condition of high perceptual load (as may occur when the objects moving at the speed of 12°/s in the Wolfe study) than in case of low perceptual load (as may occur when the objects moving at the speed of 12°/s in the present study).^[22]^ When adding or subtracting items from the tracking set at a slow speed, participants may lose targets by turning their attention to distractors, resulting in a decrease in tracking accuracy. This explanation is supported by some findings, Pylyshyn and Annan(2006) discovered that interference between the onset cue and the action of deselection could result in decreased performance for deselection but have no effect on selection.^[27]^ A second possible reason for our finding is that the speed change may has different effects on the performance of target object presentation task. In other words, as the target movement slows, the performance of the added and dynamic conditions barely improves, resulting in a larger performance gap between these 2 conditions and the fixed condition, which eventually becomes significant. Future study should consider make more investigation using techniques such as eye movement tracking.

Despite the lack of a statistically significant difference between players and non-players, an intriguing phenomenon was observed: the gap between the 2 groups gradually widened from fixed condition to added condition to dynamic condition. The current study found that the greatest difference between elite athletes and non-athletes was in dynamic condition, suggesting that expert advantage is greater in complex dynamic situations. This may be related to the characteristic of soccer, in which the environment is typically visually chaotic and requires constant target tracking. In such a setting, soccer players must be able to modify their tracking of multiple moving objects in a dynamic environment. According to the research of Furley and Wood,^[28]^ experts in team sports only differ in cognitive processing skills that are directly related to their field of expertise. Due to the resemblance between added and dynamic conditions and real-life soccer situations, greater performance is observed under these conditions. Professional athletes as a group have extraordinary abilities for rapidly learning unpredictable, complex, and context-free dynamic visual scenes.^[16]^ This tracking ability appeared to have transferred from training to the experiment task.^[22,24]^

## 10. Limitations

A few limitations must be taken into consideration. Previous studies have shown that the performance of MOT task is affected by different object velocities. Tracking accuracy decreases as the speed of object movement increases. In the present study, we used a fixed speed at 5°/s for the MOJ task. At this speed, the tracking accuracy of 3 target presentation tasks did not differ between groups, which may reflect that the level of load difficulty selected in this study was not high to distinguish the difference between groups. Future study can explore the influence of different object movement speed on MOJ task. Another limitation is that we didn’t do any press test in this study, cognitive anxiety may be affecting our results.

## 11. Conclusion

In recent years, researchers have expressed a significant level of interest in the relationship between visual tracking abilities and sporting expertise. The MOJ task offers a new metric to study the tracking behaviors of soccer players in a dynamic environment. This study indicated that adding and subtracting items from the tracked set could present a challenge for the processes supporting MOT. This may depend on the difficulty levels of tasks, such as the speed of moving objects. Furthermore, there were no significant differences in tracking accuracy between female soccer players and non-players when the targets were presented in fixed and added condition. The tracking performance of female soccer players are significantly better than non-players in dynamic condition, suggesting that experts who specialize in team sports tend to exhibit a greater attention advantage in areas that are pertinent to their field of expertise.

## Author contributions

**Conceptualization:** Qian Su.

**Data curation:** Qiang Dai, Baokun Li.

**Methodology:** Jingcheng Li, Feng Wang.

**Software:** Feng Wang.

**Writing – original draft:** Qian Su.
